# Thoracoschisis associated with Limb Body Wall Complex

**DOI:** 10.21699/ajcr.v8i3.568

**Published:** 2017-05-01

**Authors:** Dragana Vujovic, Aleksandar Sretenovic, Maja Raicevic, MarijaLukac MarijaLukac, Tamara Krstajic, Vesna Ljubic, Sanja Sindjic Antunovic

**Affiliations:** 1 Department of Pediatric Surgery, University Children’s Hospital,Belgrade, Serbia; 2 Department of Surgery and Anesthesiology, Faculty of Medicine, Universityof Belgrade, Belgrade, Serbia; 3 Department of Pediatric Surgery, Clinic for Pediatric Surgery andOrthopedics, Clinical Center Nis, Nis; 4 Department of Neonatology, General Hospital Zemun, Belgrade, Serbia

**Keywords:** Thoracoschisis, Riedel lobe, Limb deformity, Newborn

## Abstract

Thoracoschisis is a rare condition. A female newborn presented with right-sided thoracoschisis, associated with diaphragmatic hernia and protrusion of an accessory liver lobe through the chest wall defect along with deformity of the right forearm and hand duplication. Diagnosed as part of the limb-body wall complex (LBWC), management included resection of the exteriorized liver lobe followed by right hemidiaphragm and thoracic wall reconstruction.

## INTRODUCTION

Thoracoschisis is a rare congenital anomaly that usually presents an evisceration of organs through a lateral chest wall defect [1]. It is often associated with a diaphragmatic hernia, defect of the anterior abdominal wall and limb malformation, the so-called "limb-body wall complex" (LBWC) deformity [2]. The etiology of this complex anomaly is still unknown, but early embryonic developmental defects are considered as probable pathogenetic factors with a low recurrence risk. Prenatal diagnosis includes detection of high levels of maternal serum alpha-fetoprotein and thorough ultrasonographic examination at the end of the first trimester of pregnancy could indicate this severe malformation [3]. There is no correlation with parental or fetal karyotype disorders, although an association of these anomalies is almost invariably fatal. We are reporting a case of thoracoschisis associated with LBWC.

## CASE REPORT

A full-term female newborn (weight 2600 gram) was transferred to our institution for the management of the right anterior chest wall defect associated with limb abnormalities. There was herniation of a tissue, 5cm long and 3cm wide, through a thoracic wall defect approximately 4cm above the right nipple. The limb abnormality included general hypoplasia of the ipsilateral arm and with an incomplete hand duplication (Fig.1). No previously hereditary disease was reported in family and pregnancy and delivery by cesarean section was without any complications.

Ultrasound examination of the abdomen and central nervous system (CNS) showed no associated anomalies and echocardiography also excluded congenital heart malformation. Computed tomography (CT) delineated the elevation of the liver with the Riedel lobe rising from its anterior edge, herniated through the ventral defect of the right hemidiaphragm, behind the ribs and extrapleurally, exteriorizing through the first intercostal space and anterior chest wall defect in the upper third of the anterior axillary line (Fig. 2). Humerus was mostly within a preserved shape and size, only distal epiphysis had a fork-formation shape. Two short, arc curved bones of deviated right forearm were noticed, with two hands distally, one with marked deformation, but notable four metacarpal bones and several phalanges, and second, rudimentary, on the anterior side of the forearm (Fig. 3). Achieving sufficient respiratory support on mechanical ventilation, the patient was operated on the second day of life. Laparotomy was performed through the right subcostal incision. Herniated content was pulled into the abdomen and the Riedel lobe excised. Defects of the right hemidiaphragm and anterior chest wall were closed. The postoperative course was uneventful, although prolonged due to some respiratory problems. Histopathology confirmed the normal liver tissue of the Riedel lobe. The patient was discharged three weeks after surgery. During later follow-up, for another two years, the child was doing well and orthopedic prosthetic treatment is now scheduled.

**Figure:**
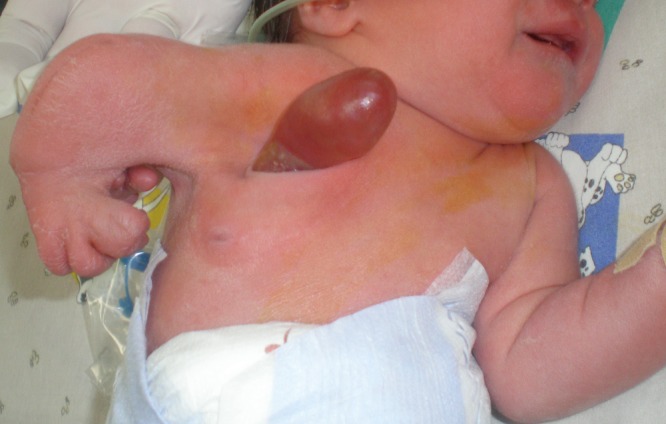
Figure 1: A) Eviscerated Riedel lobe.

**Figure:**
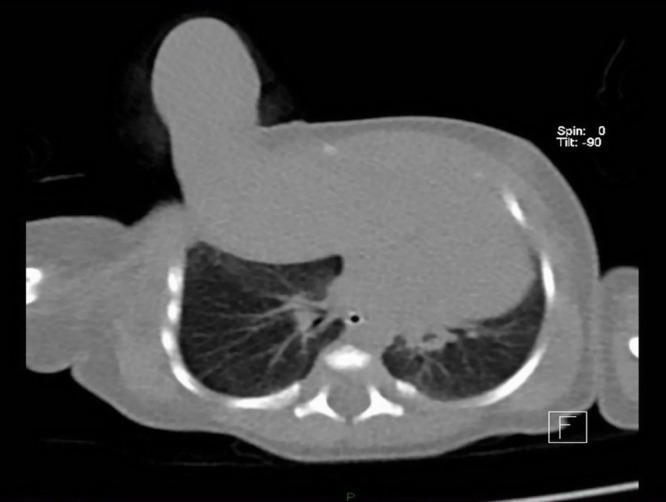
Figure 2: A) CT scan of liver showing liver protrusion through the thoracic wall defect.

**Figure:**
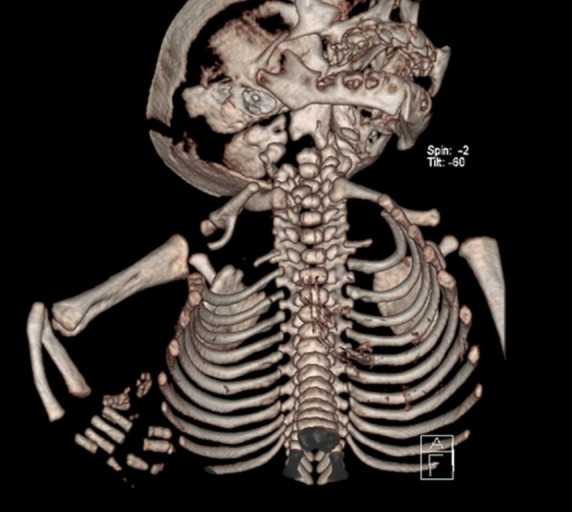
Figure 3: A)CT scan of right limb deformity.

## DISCUSSION

Thoracoschisis is an extremely rare congenital anomaly. In this condition there is thoracic wall defect lateral to the midline, through which liver and intestine can herniated after passing through the diaphragmatic defect on the same side. The liver lobe that exteriorizes throughout the thoracic wall defect is an accessory Riedel lobe. This lobe is considered as a normal variation in liver segmentation [4]. When associated with severe limb deformity, it could be regarded as part of LBWC. Van Allen et al described a LBWC deformity that consists of a poly-malformation syndrome with the presence of two out of three fetal anomalies: thoraco/abdomino-schisis associated with eventration of internal organs, craniofacial malformation and limbs deformities [5]. A literature search revealed only 11 reported cases of thoracoschisis with LBWC [1,2,6-14]. Almost all had an associated diaphragmatic hernia and limb abnormality, except the case of the only male patient with the left diaphragmatic eventration [8]. Two cases of isolated thoracoschisis with the intact diaphragm and without limb deformity were also reported in literature [10,14]. The liver was most often herniated among other abdominal organs in all cases. Eight patients had left-sided thoracoschisis. The present case is the first female right-sided thoracoschisis with accompanying diaphragmatic hernia and limb deformity who survived long-term, after surgical treatment.

## Footnotes

**Source of Support:** Nil

**Conflict of Interest:** None declared

